# Novel Non-Histocompatibility Antigen Mismatched Variants Improve the Ability to Predict Antibody-Mediated Rejection Risk in Kidney Transplant

**DOI:** 10.3389/fimmu.2017.01687

**Published:** 2017-12-05

**Authors:** Silvia Pineda, Tara K. Sigdel, Jieming Chen, Annette M. Jackson, Marina Sirota, Minnie M. Sarwal

**Affiliations:** ^1^Division of Transplant Surgery, Department of Surgery, University of California, San Francisco (UCSF), San Francisco, CA, United States; ^2^Institute for Computational Health Sciences, University of California, San Francisco (UCSF), San Francisco, CA, United States; ^3^Department of Medicine, Division of Immunogenetics and Transplantation Immunology, The Johns Hopkins University School of Medicine, Baltimore, MD, United States; ^4^Department of Pediatrics, University of California, San Francisco (UCSF), San Francisco, CA, United States

**Keywords:** kidney organ transplant, antibody-mediated rejection, exome sequencing, non-histocompatibility antigen, gene expression, machine learning

## Abstract

Transplant rejection is the critical clinical end-point limiting indefinite survival after histocompatibility antigen (HLA) mismatched organ transplantation. The predominant cause of late graft loss is antibody-mediated rejection (AMR), a process whereby injury to the organ is caused by donor-specific antibodies, which bind to HLA and non-HLA (nHLA) antigens. AMR is incompletely diagnosed as donor/recipient (D/R) matching is only limited to the HLA locus and critical nHLA immunogenic antigens remain to be identified. We have developed an integrative computational approach leveraging D/R exome sequencing and gene expression to predict clinical post-transplant outcome. We performed a rigorous statistical analysis of 28 highly annotated D/R kidney transplant pairs with biopsy-confirmed clinical outcomes of rejection [either AMR or T-cell-mediated rejection (CMR)] and no-rejection (NoRej), identifying a significantly higher number of mismatched nHLA variants in AMR (ANOVA—*p*-value = 0.02). Using Fisher’s exact test, we identified 123 variants associated mainly with risk of AMR (*p*-value < 0.001). In addition, we applied a machine-learning technique to circumvent the issue of statistical power and we found a subset of 65 variants using random forest, that are predictive of post-tx AMR showing a very low error rate. These variants are functionally relevant to the rejection process in the kidney and AMR as they relate to genes and/or expression quantitative trait loci (eQTLs) that are enriched in genes expressed in kidney and vascular endothelium and underlie the immunobiology of graft rejection. In addition to current D/R HLA mismatch evaluation, additional mismatch nHLA D/R variants will enhance the stratification of post-tx AMR risk even before engraftment of the organ. This innovative study design is applicable in all solid organ transplants, where the impact of mitigating AMR on graft survival may be greater, with considerable benefits on improving human morbidity and mortality and opens the door to precision immunosuppression and extended tx survival.

## Introduction

Chronic kidney disease (CKD) is a major public health problem. As renal function progressively declines in CKD patients over time, they progress to end-stage renal disease (ESRD), when renal replacement therapy becomes critical to conserve quality of life. For all ESRD patients, transplantation (tx) is the preferred treatment, as it provides better patient survival than prolonged dialysis therapy (1, 2). Tx occurs across histocompatibility antigen (HLA) barriers and requires life-long immunosuppression, to effectively suppress donor-specific injurious immune responses, while conserving immune recognition to foreign and infectious antigens. There are several types of graft failure. T-cell-mediated rejection (CMR) involves T-cell activation and can be effectively treated with augmentation of immunosuppressive therapies. Antibody-mediated rejection (AMR) involves B cell and plasma cell activation resulting in the generation of donor-specific antibodies (DSA), which bind to HLA and/or non-HLA (nHLA) molecules on the endothelium. The presence of pre-formed and *de novo* (newly formed) DSA, specific to D/R mismatches are major risk factors for AMR, which results in both acute and chronic tx injury and is the primary cause of accelerated early and late allograft loss (3). The major cause of untimely tx failure relates to the extent of HLA mismatch between donor (D) and recipient (R) (4, 5), with additional contributory factors such as longer period of dialysis before tx, ischemia-reperfusion injury at tx, and post-tx exposure to the fibrosing injury of a class of immunosuppressive drugs that relate to calcineurin inhibition as their mechanism of action and non-adherence to immunosuppression therapy.

The current D/R matching for organ tx approach relies on three major criteria—blood group compatibility, D/R matching at the major HLA loci for Class I (A/B/C) and Class II (DR/DP/DQ) for kidney tx, and evaluation of sensitization risk by evaluation of pre-formed antibodies to major HLA loci. HLA is a well-characterized complex locus on chromosome 6, formed by a number of genes encoding the major histocompatibility complex proteins in humans. In graft rejection, any cell displaying another HLA type may be seen as an invader by the body’s immune system resulting in the rejection of the tissue/organ bearing those cells. Therefore, it is clear that HLA mismatch represents an important risk factor for kidney graft rejection after tx (6).

Recently, mismatched nHLA antigens between the D/R have also been recognized to drive immunogenicity and tx rejection (7–9). In fact, the important role of mismatched nHLA antigens in driving graft injury can be specifically recognized given that acute rejection can occur even in very well HLA matched and even HLA-identical kidney tx (10, 11). Unfortunately, specific nHLA immunogenic antigenic D/R mismatches, which may increase the risk of rejection after tx, are difficult to predict, and are as yet, poorly defined.

The publication of human genome data and the availability of novel tools for high-dimensional sequencing of DNA in donors and recipients presents an unprecedented opportunity to improve the D/R matching in organ tx and to extend this analysis to both major and minor (nHLA) HLA epitopes. Genetic association studies of candidate genes that are linked to tx rejection have reported single-nucleotide polymorphisms (SNPs) in genes encoding cytokines, chemokines, toll-like receptors, and VEGF (12). Population-based assessments suggest a familial component to rejection-free tx course (13) and a recent GWAS study (14) identified two loci (PTPRO and CCDC67) as associates with a specific phenotype of CMR. In addition, amino acid mismatches in transmembrane proteins in D/R pairs (15) were identified to be a predictor of long-term graft function in kidney tx recipients, further highlighting the critical role of nHLA immunogenic epitopes in the organ tx. There have been no published studies that have systematically identified D/R nHLA epitopes that can be predictive of risk of AMR and CMR after tx. Identifying different rejection subtypes is important as the underlying biological drivers are likely different, and effective resolution of each type of rejection episode requires disparate treatments. Treatment of CMR is well defined with the use of corticosteroids and T cell depleting agents (16), but effective treatments for AMR remain to be identified, despite some efficacy for AMR resolution from plasmapheresis, intravenous immune globulin, and B cell depletion (17).

The explosion of advanced computational and statistical methods, combined with advances in sequencing technologies in the recent years, allowed us to carry out integrative translational research to address the identification of relevant D/R non HLA variants prior to tx, that can predict the risk of post-tx injury and, thus, advance on current methods of tx patient risk stratification in clinical practice. In this report, we conducted a focused study of exome sequencing (exomeSeq) of DNA samples from a set of kidney tx D/R pairs and performed statistical analyses to identify an association between the mismatched genetic variants by D/R pairs and biopsy-confirmed clinical outcome of rejection (either AMR or CMR) and no-rejection (NoRej) after kidney tx. The results of this study provide a fingerprint of D/R variants to stratify recipient risk of AMR or CMR after kidney tx, with respect to the intended donor organ, prior to the actual tx, opening the door for precision D/R selection and tx immunotherapy.

## Materials and Methods

### Study Design

**Figure 1:**
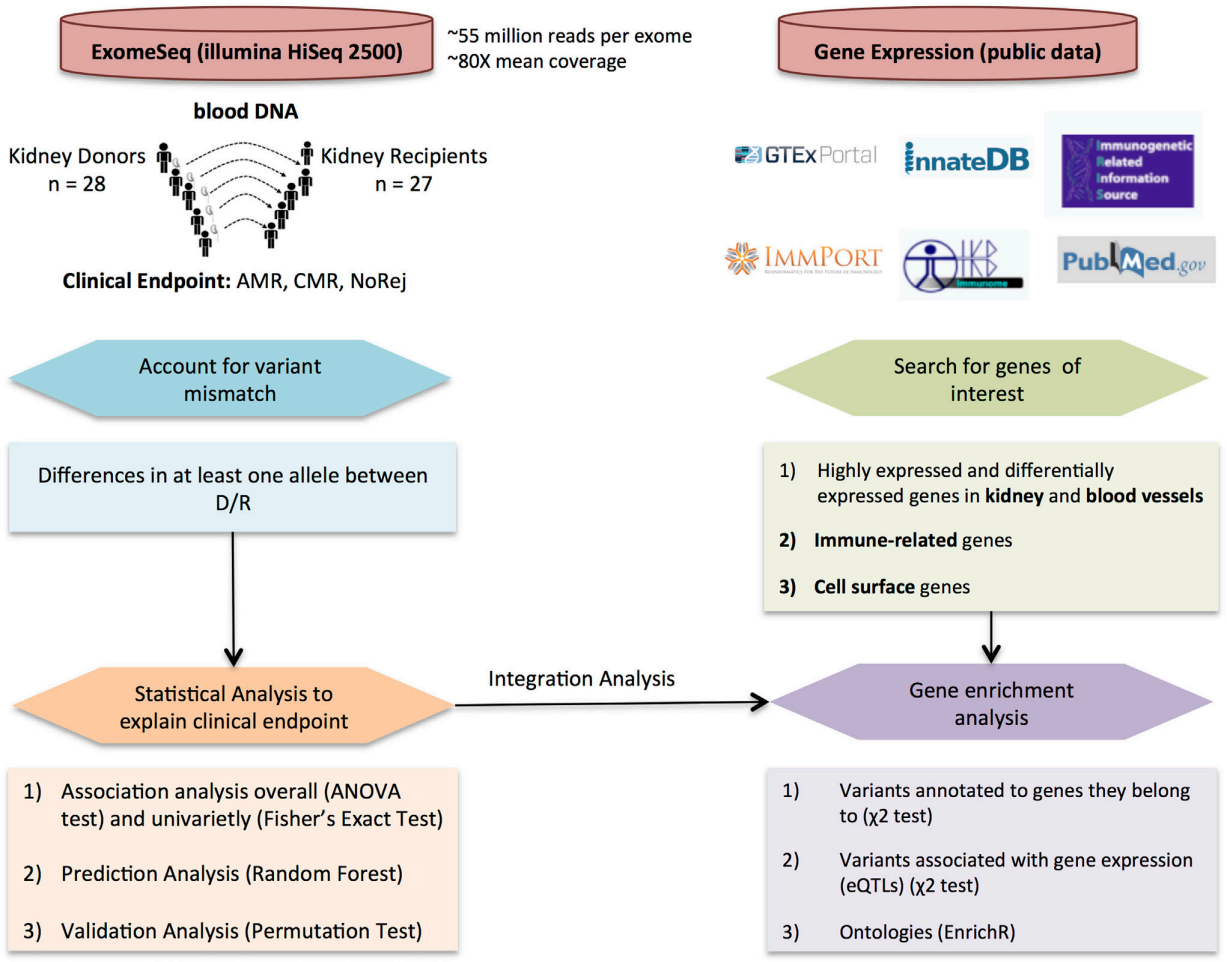
Overall study design and workflow. Detection of variant mismatches and statistical analysis for association with clinical endpoints and prediction using exome sequencing (exomeSeq) data (left panel) integrated with the analysis of publically available gene expression data (right panel) to perform enrichment analysis using different variant annotations.

Fifty-five individuals paired by D/R from 27 kidney tx from 28 kidney donors (one recipient had to go through a second tx) were selected from John Hopkins, obtained as an institutional IRB approved study (in keeping with the guidelines in the Declaration of Helsinki) and sequenced using blood DNA. Each blood sample was obtained from the donor and the recipient prior to tx. Recipients were selected in one of three clinical categories based on the presence or absence of biopsy-proven rejection in the first 6 months after tx. There were 14 recipients confirmed with AMR, seven recipients confirmed with CMR, and seven stable recipients without rejection. Patients with normal 6-month protocol biopsies and stable graft function based on the evaluation of the serum creatinine were grouped in the NoRej group. Patients with biopsy-confirmed acute rejection, based on an indication biopsy for graft dysfunction (>20% rise in the serum creatinine above baseline) were classified into either CMR or AMR based on standardized Banff classification of kidney allograft histopathology (18–20). To enrich for patients with post-transplant biopsy-confirmed rejection in the first 6 months, we selected patients who were highly sensitized (mean cPRA 47 ± 45). Twelve of the 14 recipients in the AMR cohort tested positive for DSA at the time of transplant and 13 of 14 tested positive for DSA at time of biopsy. Patients received thymoglobulin for induction and were maintained on steroids, tacrolimus, and mycophenolate mofetil for their maintenance immunosuppression regimen. To enrich for patients with post-transplant biopsy-confirmed rejection in the first 6 months, we selected patients who were highly sensitized (mean cPRA 47 ± 45). Molecular HLA typing was performed by reverse sequence specific oligonucleotide hybridization (LABType, One Lambda, Canoga Park, CA, USA). Donor-specific HLA antibodies were evaluated using solid-phase immunoassays (Lifecodes classes I and II ID panels; Immucor-Lifecodes, Stamford, CT, USA; Single Antigen Beads; One Lambda, Canoga Park, CA, USA) performed on a Luminex platform. Unacceptable HLA antigen assignments and CPRA calculations were based on HLA antibody specificities strong enough to yield a positive flow cytometric crossmatch. In this cohort, we have integrated exomeSeq and clinical data with functionally relevant gene expression data leveraging selected publically available datasets. The overall design is showed in Figure 1.

### DNA Extraction and exomeSeq

DNA was extracted from PBMCs collected from donor and recipients using EZ1^®^ Advanced XL automated DNA extractor from Qiagen and EZ1 DNA Blood 350 µl Kit (Cat No./ID: 951054, Qiagen Inc.). The DNA was measured using NanoDrop 2000 Spectrophotometer (Thermo Fisher Scientific). We used Kapa Hyper Library Prep kit (KAPA BIOSYSTEMS) for making the libraries and SeqCap EZ Human Exome Kit v3.0 (Roche Sequencing) for the exome capture. The exome captured libraries were then sequenced on the HiSeq 2500 for paired-end 100 bp on the High Output mode. We performed exomeSeq on the 55 DNA blood samples using Illumina HiSeq 2500 with an average of 55 million reads per exome and mean coverage of 80×. Raw data were aligned to the human genome build 37 (hg19) using bwa-mem (0.7.15) (21).^1^ Fastqc (0.11.5) was used as a quality control tool for the sequence data. Picard (1.141) was used for marking duplicates in the bam file. We used the Genome Analysis Toolkit (GATK) (3.4-46) (22) to perform the subsequence analysis. This is a software package for analysis of high-throughput sequencing data. GATK’s BaseRecalibrator was used to generate recalibrated and realigned bam files. GATK’s HaplotypeCaller was used for the variant calling and the filtering was done using variant quality score recalibration according to GATK Best Practices recommendations (23, 24). The variant recalibrator evaluates variants in a two-step process, each performed by a distinct tool: (1) VariantRecalibrator: create a Gaussian mixture model by looking at the annotations values over a high-quality subset of the input call set and then evaluate all input variants. This step produces a recalibration file. (2) ApplyRecalibration: apply the model parameters to each variant in input VCF files producing a recalibrated VCF file in which each variant is annotated with its VQSLOD value. In addition, this step will filter the calls based on this new VQSLOD score by adding lines to the FILTER column for variants that do not meet the specified VSQLOD threshold. We additionally excluded the multiallelic SNPs and insertions and deletions (indels). We finally annotated the variants using ANNOVAR (25) identifying a total number of 515,899 variants restricted to the autosomal chromosomes. From these variants, we only considered the variants that were called in at least 95% of the samples, resulting in a total of 488,539 variants for subsequent analyses.

### D/R Variant Mismatch

**Figure 2:**
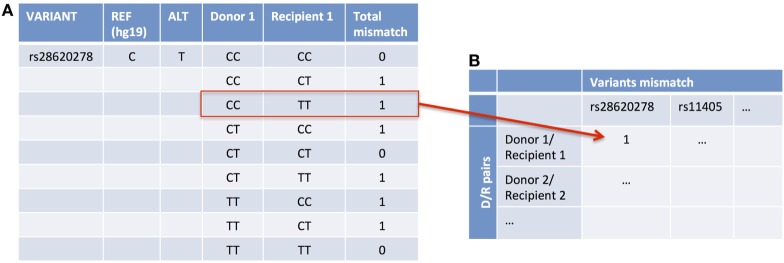
An example of accounting for variant mismatch using one example of a D/R pair and one variant. In this case, the Donor 1 is CC and Recipient 1 is TT **(A)**, so it is considered as a mismatch and, therefore, it is assigned as a 1 in the data matrix **(B)**.

We measured the variant mismatch between D/R pairs considering one allele difference in at least one of the individuals. An example of all the possible allele combinations that one pair may have for one specific variant aligned to the reference genome and the total mismatch is represented in Figure 2A. The data matrix for the analysis will account for the mismatch considering all the variants and D/R pairs (Figure 2B). With the total number of mismatches, we performed an ANOVA-test to account for the global association with the clinical endpoints (AMR, CMR, or NoRej) adjusting the model by a “genomic distance,” which takes into account the race and relatedness information of each D/R pair. We obtained the genomic distance by assessing the first two principal components in a principal component analysis (PCA) with the 1000 Genomes Project panel and obtaining the Euclidean distance by pairs.

### Association Analysis Considering Clinical Endpoints

We assessed the association of the variants with the clinical endpoints. Using a data matrix with the 28 D/R pairs as columns and all the variants with at least one pair mismatched (472,400 variants) (Figure 2B) as rows, we applied Fisher’s exact test to find an association between each specific mismatched variant and the clinical endpoints. To find the mismatched variants that are associated with an increased risk of AMR and/or CMR, we looked to the number of variants of which the number of pairs was higher in each group in comparison to the others.

### Creating Gene Sets of Interest

We leveraged publicly available datasets to functionally annotate our variants and genes of interest. We proposed four lists of genes of interest: (1 and 2) Genes highly expressed and differentially expressed (DE) in kidney and blood vessels, (3) genes that are immune related, and (4) genes that are expressed on the cell surface. To find DE genes in kidney and blood vessels, we used the data processed by the TOIL project (26) from the GTEx Consortium.^2^ They have recomputed and processed RNA-Seq samples to create a consistent meta-analysis of four datasets (GTEx, TCGA, TARGET, and PNOC) free of computational batch effects. We used the normalized counts they provided from the RSEM algorithm to find genes that were upregulated and/or highly expressed in kidney and blood vessels. To find genes DE and upregulated, we used Wilcoxon rank test and applied Benjamini and Yekutieli (27) FDR for multiple testing (MT) correction. We considered all the genes that were DE, compared to all the remaining tissues and to each of the remaining tissues. For the highly expressed genes, we considered all the genes of which the mean was higher that the mean of all genes plus 1 SD. We ended up with a list of 2,786 kidney genes and 3,291 blood vessel genes.

To find immune-related genes, we downloaded four lists of genes through the innateDB webpage^3^ containing a total of 8,745 genes. We used the gene list provided by ImmPort (28), Immunogenetic Related Information Source (IRIS) (29), Immunome Database (30), and the innate immune response curated by innateDB (31).

Finally, we looked for cell-surface genes extracted from publically available data resulting in a list of 3,845 genes. This list was established by considering the genes that appear in the following protein/peptide databases including the HUPO Plasma Proteome Project (32), a non-redundant list from the Plasma Proteome Institute (33), the MAPU Proteome database (34), and the Surfaceome (35).

### Gene Enrichment Analysis

To perform the gene enrichment analysis, we first annotated the variants associated with AMR to genes in two different ways: (1) considering the genes they are located in and (2) considering the eGenes from the expression quantitative trait loci (eQTL) analysis from GTEx in blood vessels and whole blood. Considering these two annotations for the variants, we performed an enrichment analysis using a X^2^-test with the four lists of genes (kidney, blood vessels, immune related, and cell surface).

Finally, to provide a biological interpretation to the results, the annotated variants were analyzed with the EnrichR tool, an integrative web-based tool that performs enrichment analysis providing various types of visualization summaries of collective functions of gene lists.^4^

### Prediction Analysis of Clinical Endpoints

We applied random forest (RF) to overpass the MT correction problem and a lack of statistical power. RF is a machine learning technique for prediction and classification problems that works well with small sample size and uses the generation of several random trees to avoid the detection of false positives and over-fitting. RF was proposed by Breiman in 2001 (36) and is an appropriate method for our problem since it can be used when the sample size is much smaller than the number of variables (28 pairs ≫ 472,400 variants) and it allows a multi-class classification. RF does not perform a variable selection by itself, so we applied the R package variable selection method using RF (VSURF) (37) that proposes a variable selection method based on RF by minimizing the out of bag error (OOB) rate. In order to find a specific subset of variants that classify our samples based on the clinical endpoints. In RF, there is no need for cross-validation or a separate test set to get unbiased estimates since each tree is built using a bootstrapped sample from the original data. One-third of the cases are left out from the construction of the tree and it is used as a test set to obtain the OOB error. Nevertheless, we generated 10 permuted datasets by shuffling the clinical endpoints from the original data and applied the same algorithm with VSURF.

## Results

The aim of this study was to discover the impact of mismatched genetic variants between D/R kidney tx pairs associated with three different clinical endpoints in recipients after tx with their specific donor organs. These different clinical endpoints distributed 28 recipients in one of three categories: (1) NoRej group (*n* = 7): stable graft function (stable serum creatinine) and protocol biopsy-confirmed absence of any significant pathology or rejection; (2) CMR group (*n* = 7): graft dysfunction (>20% increase in serum creatinine from baseline) and biopsy-confirmed CMR using Banff criteria (38); and (3) AMR group (*n* = 14): graft dysfunction and biopsy-confirmed antibody-mediated rejection using Banff criteria (19), with or without DSA to major HLA antigens. To perform analysis of D/R variant mismatching, we carried out exomeSeq on the 28 D/R pairs prior to kidney organ tx. All patients were on similar maintenance immunosuppression with mycophenolate mofetil, tacrolimus, and steroids, and induction with thymoglobulin. Demographic parameters, inclusive of cause of ESRD, were matched among the three subsets of patients (Table 1).

**Table 1:** Demographics for the donors and recipients samples.

	Antibody-mediated rejection (*n* = 14)	T-cell-mediated rejection (*n* = 7)	No-rejection (*n* = 7)
Recipient gender (Female, %)	10 (71)	4 (57)	6 (86)
Recipient age (years)	54.2 ± 16.2	47.3 ± 14.9	59.4 ± 13.9
Recipient race (Caucasian/African-American)	7/7	6/1	5/2
Donor gender (Female, %)	6 (47)	4 (57)	3 (33)
Donor age (years)	49.2 ± 14.1	48.0 ± 16.5	55.0 ± 7.0
Donor race (Caucasian/African-American)	13/1	6/1	6/1
Type of transplant (%)-living	6 (43)	2 (29)	1 (14)
Type of transplant (%)-living related	2 (14)	4 (57)	5 (72)
Type of transplant (%)-cadaver	6 (43)	1 (14)	1 (14)
% PRA (mean ± SD)	55 (45)	34 (47)	41 (46)
Donor-specific antibodies at time of transplant (±)	12/2	6/7	4/7

The total number of D/R variant mismatches assessed prior to tx was noted to be significantly higher in the AMR group (ANOVA-test, AMR vs. NoRej, *p*-value = 0.02). Additional analysis of specific D/R variant mismatches that specifically associate with one or more clinical endpoints, identified a novel set of 123 variants (Fisher’s exact test, *p*-value <0.001). A minimal set of 65 variants (from the set of 123 variants) was selected with RF (accuracy error = 0.03) and provided clean classification of all three-sample phenotype outcomes for the recipients after tx, with very robust performance on repeated permutation testing. We also leveraged publically available gene expression data as selection filters for narrowing the most informative variant list to map to genes that are highly enriched in the transplanted organ (kidney), to the anatomical site most affected by AMR (blood vessels), to select for candidates enriched in the rejection process (immune related), and to select candidates that are more likely to be recognized by the recipients’ immune response (cell-surface expression). Figure 1 summarizes the overall study design.

### Higher Number of Pre-tx D/R Mismatched Variants Associate with Increased Risk of Post-tx AMR

**Figure 3:**
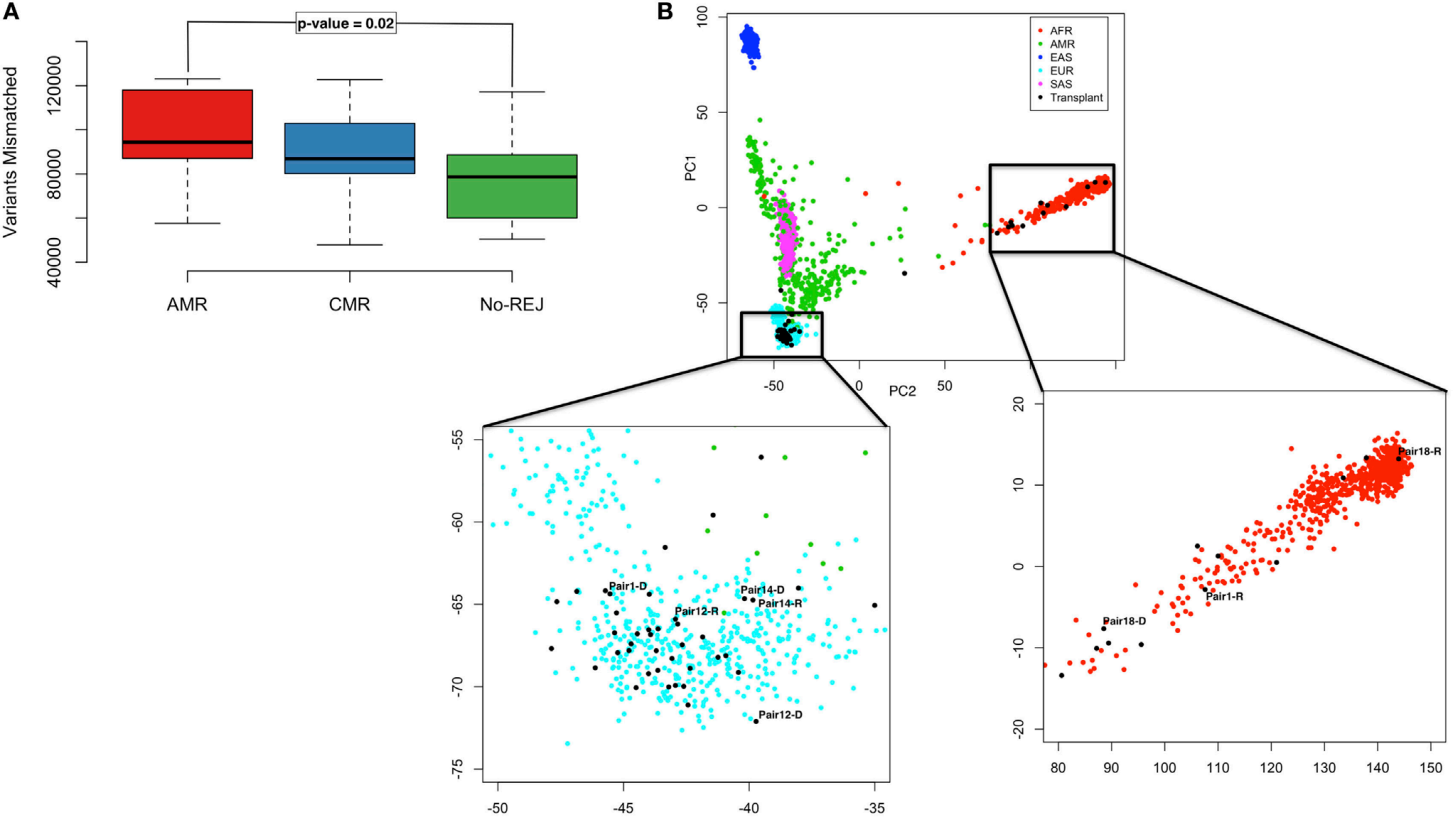
Boxplot representing the distribution of mismatched variants in each D/R pair, stratified by the clinical endpoints of antibody-mediated rejection (AMR), T-cell-mediated rejection (CMR), and no-rejection (NoRej) **(A)**. Principal component analysis plot with the 1,000 G as reference panel (AFR, African population; AMR, American population; EAS, East Asian population; EUR, European population; and SAS, South Asian population). The black dots represent the 28 pairs from our data. Pair 1 shows an example of race mismatch, Pair 14 an example of pair related, and Pairs 12 and 18 an example of a white pair and a black pair, respectively **(B)**.

The variant differences per D/R pair were evaluated with respect to the human reference genome build 37 (hg19). Variant mismatches were considered if one of the alleles between the donor and the recipient at a particular SNP position was different. We identified 472,400 variants that were mismatched in at least one D/R pair: 386,958 had at least one mismatch in the AMR group, 268,722 in the CMR group, and 248,531 in the NoRej group. In Figure S1 in Supplementary Material, we show the number of mismatched variants per D/R pair with information on race mismatch and relatedness. We observed a significantly increased number of mismatched variants in the AMR group in comparison with the NoRej group (Figure 3A) (ANOVA *p*-value = 0.04), but as expected, we also observed that the number of mismatched variants was also dependent on race differences between the donor and the recipient and whether the D/R pair was related. As the AMR group was noted to have the largest number of D/R race mismatches, we explored the relative impact of AMR and race mismatches on the number of variant mismatches in each D/R pair by performing a PCA using genomic data from the 1000 Genomes Project (39) as a reference panel (Figure 3B). As expected, the donors and recipients from our study clustered further or closer according to the number of mismatched variants, and also clustered together with the population that was consistent with their self-reported race (Figure 3B). Some informative examples highlighted here are pair 1 (119,733 mismatched variants)—the donor is white self-reported and the recipient is black self-reported and are seen to cluster in the PCA plot with the Caucasian population (pair1-D) and the African population (pair1-R); pair 14 (51,058 mismatched variants)—both cluster closely with the Caucasian population and are siblings; pair 12 (90,170 mismatched variants)—both are white self-reported—not related and are seen to cluster with the Caucasian population but further than pair 12, that are related; and finally, pair 18 (123,100 mismatched variants)—both D/R are African and have the highest number of mismatched variants, which highlights much higher variability within the African population in comparison with the Caucasian. To allow for these observed differences by relatedness and race, we accounted for genomic distance considering the Euclidean distance in the plot by D/R pair. We used this variable to adjust the previous ANOVA analysis and observed that AMR was still significantly associated with a significantly higher number of mismatches (*p*-value = 0.02; AMR vs. NoRej).

To evaluate the biological significance of the observed mismatched variants, we next examined their functional classification. 25% of the mismatched variants were exonic with almost half of them being non-synonymous and thus more likely to have an impact on protein function. We applied the same ANOVA-test considering only the non-synonymous variants and similar to the previous results, these were found to be significantly higher for the D/R pair where the recipient went on to develop AMR after tx (Figure S2 in Supplementary Material).

### D/R Mismatched Variants Are Associated with AMR after Transplantation

**Figure 4:**
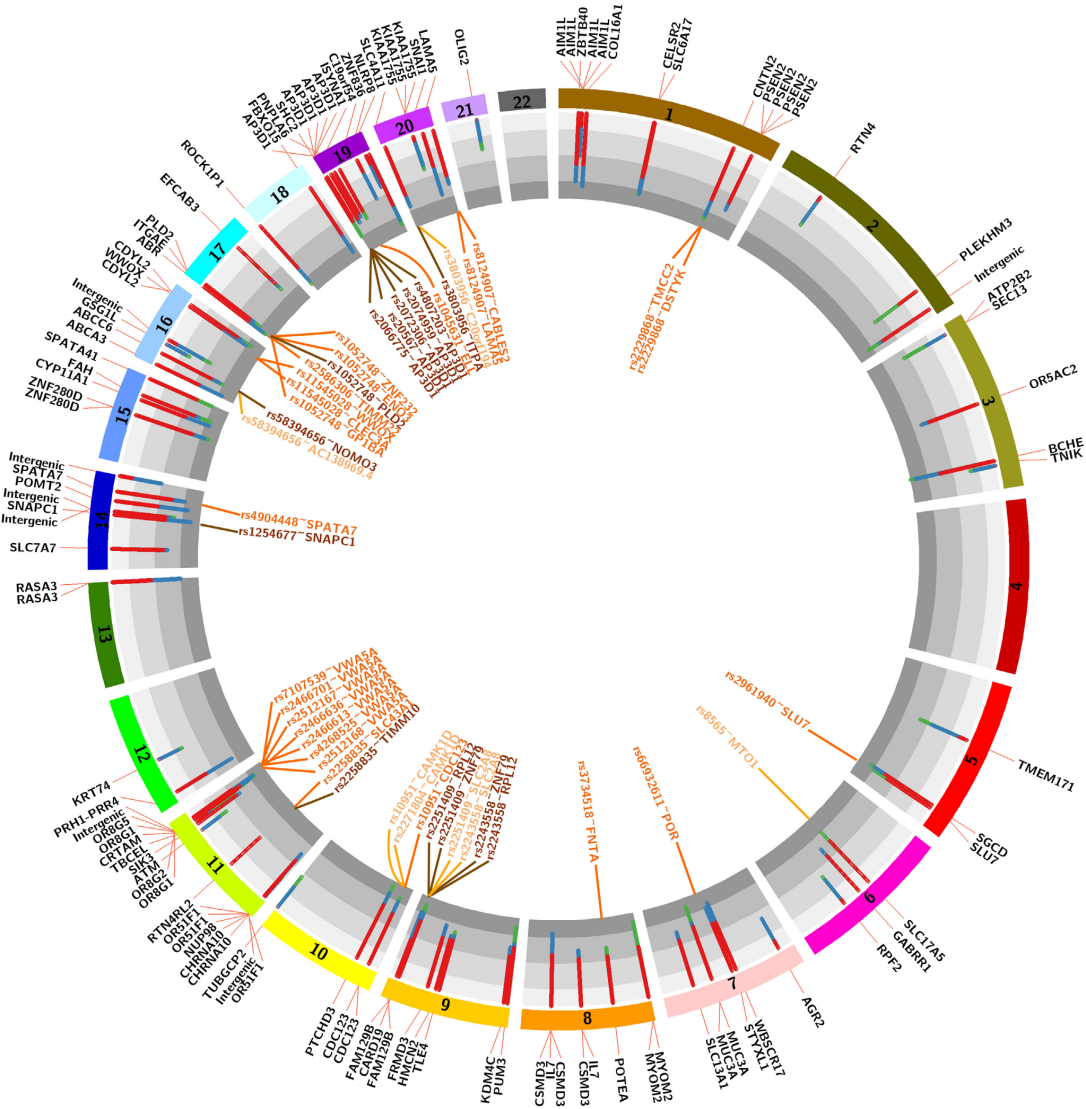
Circos plot showing the 22 autosomal chromosomes. The histogram shows the distribution of mismatches by pairs in each 123 variants associated with the three clinical endpoints (red-antibody-mediated rejection, blue-T-cell-mediated rejection, and green-no-rejection). The genes outside represent the harbor gene (black text) and the genes outside represent the expression quantitative trait loci (eQTLs; light orange-eQTLs in whole blood, dark orange-QTLs in blood vessels and medium orange-overlap between both).

123 unique variants (19 non-synonymous) (*p* < 0.001; Fisher’s exact test) were identified as nominally associated with either of three clinical endpoints of AMR, CMR, or NoRej after tx, with an incidence of 87% in the AMR, 57% in the CMR, and 20% in the NoRej. To best assess the most significant variants for each clinical group, we evaluated the maximal impact of variant sets for each D/R pair cohort, in comparison to the other two; again, we noted (as seen earlier by global analysis) an enrichment of mismatched variants for AMR, with 94 variants most enriched for AMR (AMR > CMR > NoREJ), 25 variants for CMR (CMR > NoRej > AMR), and 4 variants enriched for low immune risk and NoRej (NoRej > AMR > CMR). To account for the independence of race mismatch and relatedness between the D/R pairs, we tested if the 123 variants were associated with any of these two variables using Fisher’s exact test and none were significant corroborating the independence. Figure 4 shows the location of the variants in the genome and the percentage of mismatches within each clinical endpoints in a circos plot (40). A summary table with the information of all the variants is shown in Table S1 in Supplementary Material.

### D/R Mismatched Variants in the HLA Region Have Less Impact on Post-tx AMR than Mismatched nHLA Variants

**Figure 5:**
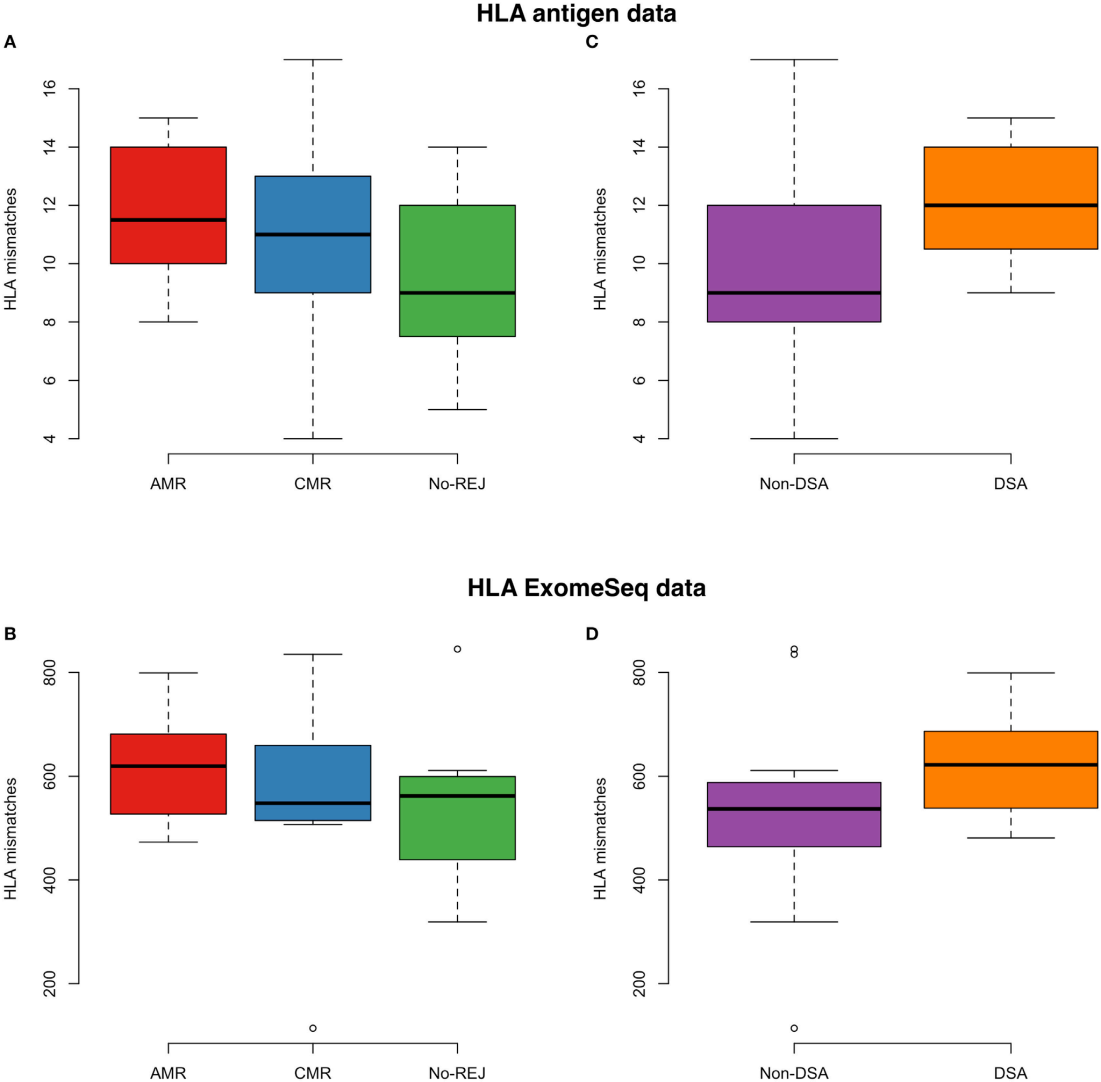
On the left panel, the boxplot represents the association analysis between histocompatibility antigen (HLA) mismatches and clinical endpoints for antibody-mediated rejection (AMR), T-cell-mediated rejection (CMR), and no-rejection (NoRej) using low-resolution HLA antigen equivalents **(A)** and HLA exome sequencing (exomeSeq) variants **(B)**. On the right panel, the boxplot represents the association analysis between HLA mismatches and presence of HLA-DSA at time of rejection using HLA antigen equivalents **(C)** and HLA exomeSeq variants **(D)**.

None of the 123 variants identified (above) belong to the HLA region. To address the potential role of HLA mismatches in these samples, we performed an association analysis between the HLA mismatches considering nine major HLA genes (*HLA-A, HLA-B, HLA-C, HLA-DRB1, HLA-DRB3, HLA-DRB4, HLA-DRB5, HLA-DQB1, HLA-DPB1*) with clinical endpoints and presence of DSA. HLA measures by serotype and exomeSeq showed highly concordant results. We performed this analysis considering the data measured by the antigens detected by HLA serotyping (standard of care) and accounting the number of variants mismatches in these nine HLA genes (Figure 5). We did not observe significant results for the association of HLA with the clinical endpoints of rejection or no rejection after tx (*p*-value = 0.3, HLA antigen data; *p*-value = 0.6 HLA exomeSeq data), though in both cases, there is a trend for a higher number of HLA mismatches in the rejection group. As expected, a higher number of DSA had borderline significance with a higher number of HLA mismatches (*p*-value = 0.07, HLA antigen data). In addition, as a positive control for the data analysis, we conducted an association analysis between HLA mismatches, race mismatch and relatedness, and as expected, we found significantly decreased number of HLA mismatches in related D/R pairs (*p*-value = 0.03) and a non-significant increase number of HLA mismatches in race mismatched D/R pairs (Figure S3 in Supplementary Material).

### The Variants Associated with Increase Risk of Post-tx AMR Are Enriched in Relevant Gene Sets

To assess the biological impact of the 123 significantly associated mismatched variants we evaluated the impact of the variants on different gene expression datasets (Figure 4). Our first assumption is that a mutation in the corresponding gene would result in a mutated mRNA and consequently a mutated protein in the donor or recipient kidney, which can trigger an antibody response in the recipient resulting in renal allograft rejection and injury. Our second assumption is that a mutation in the gene would result in different mRNA expression (eQTL) in the same gene (cis) or at another locus (trans), which would then produce a change in the expression of a protein in the donor kidney, consequently trigger an antibody response in the recipient, and drive renal allograft rejection and injury. With this in mind, we annotated the variants to genes using the eQTL analysis from GTEx (41). The GTEx Consortium has studied several tissues, but we only considered the relevant eQTLs in whole blood and blood vessels, since the key pathobiology of AMR injury in the donor kidney occurs in the donor microvasculature. Kidney tissue eQTLs are also found in the GTEx dataset, and though interesting to analyze, were not included as the number of samples with kidney tissue in GTEx was too small to perform eQTL analysis. The 94 variants associated with AMR were found to reside in 72 unique genes, as some genes had more than one variant, a factor that may weight their biological relevance. Genes with multiple variants are *AP3D1* (5 variants—1 synonymous), *CDC123* (2 variants), *CDYL2* (2 variants), *CSMD3* (3 variants), *FAM129B* (2 variants), IL7 (2 variants), *MUC3A* (2 variants—1 non-synonymous, 1 synonymous), *MYOM2* (2 variants), *OR51F1* (4 variants—2 nonsynonymous), *OR8G1* (2 variants), *OR8G5* (2 non-synonymous variants, 1 synonymous), *PNPLA6* (2 variants), *PSEN2* (4 variants—1 synonymous), *RASA3* (2 variants), *ZNF280D* (2 variants—1 non-synonymous), and the *SLC family* (5 variants—1 non-synonymous, 2 synonymous). Interestingly, we found that 7 out of the 19 non-synonymous variants (37%) were located in AMR specific genes. The 25 variants associated with CMR resided in 22 unique genes, the following of which had multiple variants: *AIM1L* (4 non-synonymous variants), *CHRNA10* (2 non-synonymous variants) and *KIAA1755* (3 variants—1 non-synonymous, 1 synonymous). For CMR, 7 out of 19 (37%) non-synonymous variants were located in genes with multiple variants. A summary table with information about these variants is shown in Table 2.

**Table 2:** Summary table with information about the variants that reside in genes with more than one associated variant.

Single nucleotide polymorphism	Chr	Position	Ref	Alt	GeneName	GeneFunction	AF_all	AF_afr	AF_eur	Diff_AMR	Diff_CMR	Diff_NoRej	*p*-Value	eGenes—blood vessels	eGenes—whole blood
rs2072306	19	2109019	T	C	AP3D1 (kidney, blood vessels, cell-surface)	Intronic	0.33	0.49	0.20	11	0	1	4.73E−04	AP3D1	AP3D1
rs20567	19	2110746	G	A		Exonic-synonymous	0.33	0.49	0.20	11	0	1	4.73E−04	AP3D1	AP3D1
rs2074959	19	2111649	T	C		Intronic	0.33	0.49	0.20	11	0	1	4.73E−04	AP3D1	AP3D1
rs2066775	19	2115493	A	G		Intronic	0.33	0.49	0.20	11	0	1	4.73E−04	AP3D1	AP3D1
rs4807203	19	2127272	A	G		Intronic	0.34	0.54	0.20	11	0	1	4.73E−04	AP3D1	AP3D1

rs2271804	10	12252217	G	A	CDC123	Intronic	0.38	0.19	0.52	11	2	0	8.08E−04		CAMK1D
rs10951	10	12292344	A	G		UTR3	0.65	0.73	0.60	13	3	1	9.08E−04	CDC123	CAMK1D

rs9940301	16	80641906	G	A	CDYL2	Intronic	0.38	0.76	0.23	13	1	3	9.08E−04		
rs9933302	16	80641931	T	C		Intronic	0.41	0.84	0.23	13	1	3	9.08E−04		

rs55980973	8	113655644	A	T	CSMD3 (cell-surface)	Intronic	0.56	0.81	0.44	12	4	0	6.82E−04		
rs6992564	8	113662299	T	G		Intronic	0.56	0.81	0.44	12	4	0	6.82E−04		
rs7839990	8	113697567	A	G		Intronic	0.56	0.78	0.45	12	5	0	3.51E−04		

rs2243558	9	130289615	C	G	FAM129B (kidney, blood vessels)	Intronic	0.58	0.59	0.68	14	1	2	1.70E−05	ZNF79, RPL12	SLC2A8, ZNF79, RPL12
rs2251409	9	130286150	A	G		Intronic	0.57	0.60	0.68	14	2	2	1.77E−04	ZNF79, RPL12	SLC2A8, ZNF79, RPL12

rs13264965	8	79672953	A	G	IL7 (immune, cell-surface)	Intronic				11	2	0	8.08E−04		
rs4739138	8	79673952	T	C		Intronic	0.21	0.34	0.07	11	2	0	8.08E−04		

rs78118592	7	100550837	C	T	MUC3A	Exonic-nonsynonymous	0.06	0.05	0.10	13	1	4	8.73E−04		
rs200242471	7	100550841	C	A		Exonic-synonymous				13	1	4	8.73E−04		

rs3817699	8	2024437	C	T	MYOM2	Intronic	0.64	0.89	0.57	12	0	6	2.74E−04		
rs3817700	8	2024446	C	T		Intronic	0.64	0.89	0.56	12	0	6	2.74E−04		

rs1030723	11	4790471	G	A	OR51F1(cell-surface)	Exonic-nonsynonymous	0.17	0.44	0.13	11	2	0	8.08E−04		
rs11033793	11	4790474	T	C		Exonic-nonsynonymous	0.17	0.44	0.13	11	2	0	8.08E−04		
rs10836609	11	4791178	C	A		Upstream	0.12	0.28	0.13	11	2	0	8.08E−04		
rs10836610	11	4791181	T	G		Upstream	0.12	0.28	0.13	11	2	0	8.08E−04		

rs4268525	11	124121199	G	C	OR8G1 (cell-surface)	Exonic-unknown	0.44	0.32	0.55	13	3	1	9.08E−04	VWA5A	
rs2466636	11	124134552	C	T		Intronic	0.44	0.29	0.55	13	3	1	9.08E−04	VWA5A	

rs2512168	11	124135009	G	A	OR8G5 (cell-surface)	Exonic-nonsynonymous	0.44	0.29	0.55	13	3	1	9.08E−04	VWA5A	
rs2512167	11	124135438	G	A		Exonic-nonsynonymous	0.44	0.29	0.55	13	3	1	9.08E−04	VWA5A	
rs2466701	11	124135481	C	T		Exonic-synonymous	0.44	0.29	0.55	13	3	1	9.08E−04	VWA5A	

rs577219	19	7615585	T	G	PNPLA6	Intronic	0.67	0.57	0.72	12	1	0	5.05E−05		
rs574663	19	7614677	C	T		Intronic	0.67	0.55	0.72	11	1	0	4.73E−04		

rs11405	1	227069677	T	C	PSEN2 (immune, cell-surface)	Exonic-synonymous	0.74	0.83	0.80	12	2	0	2.00E−04		
rs2236910	1	227073410	G	C		Intronic	0.74	0.83	0.80	12	2	0	2.00E−04		
rs2802267	1	227078955	T	C		Intronic	0.72	0.78	0.80	11	2	0	8.08E−04		
rs10753428	1	227081622	A	G		Intronic	0.70	0.80	0.80	12	2	0	8.08E−04		

rs4074317	13	114747187	G	C	RASA3	Downstream	0.65	0.64	0.74	9	7	0	1.70E−04		
rs2274716	13	114781868	G	A		Intronic	0.35	0.35	0.27	9	7	0	1.70E−04		

rs12706498	7	122788665	G	A	SLC13A1	Intronic	0.21	0.06	0.30	11	2	0	8.08E−04		
rs3734518	6	74304415	G	C	SLC17A5	UTR3	0.17	0.21	0.30	13	0	3	3.82E−05		MTO1
rs3803956	20	3214581	C	T	SLC4A11	Exonic-synonymous	0.17	0.22	0.18	12	3	0	3.99E−04	ITPA	ITPA, C20orf194
rs12737742	1	110709720	G	A	SLC6A17	Exonic-nonsynonymous	0.25	0.03	0.47	12	5	0	3.51E−04		
rs1061040	14	23242828	T	C	SLC7A7 (kidney, cell-surface)	Exonic-synonymous	0.79	0.45	0.90	12	1	0	5.05E−05		

rs28620278	15	56959028	C	T	ZNF280D	Exonic-nonsynonymous	0.53	0.71	0.41	14	3	1	4.27E−05		
rs12911191	15	56961272	T	A		Intronic	0.46	0.28	0.59	13	4	1	8.73E−04		

rs12562454	1	26671084	G	T	AIM1L (cell-surface)	Exonic-nonsynonymous	0.18	0.15	0.19	3	7	0	1.43E−04		
rs57268417	1	26671248	A	G		Exonic-nonsynonymous	0.18	0.15	0.19	3	7	0	1.43E−04		
rs11247924	1	26673076	G	A		Exonic-nonsynonymous	0.18	0.15	0.19	3	7	0	1.43E−04		
rs11247925	1	26673108	C	T		Exonic-nonsynonymous	0.18	0.15	0.19	3	7	0	1.43E−04		

rs2231547	11	3687626	T	G	CHRNA10 (cell-surface)	Exonic-nonsynonymous	0.02	0.00	0.07	0	5	1	8.18E−04		
rs2231546	11	3687651	C	T		Exonic-nonsynonymous	0.02	0.00	0.07	0	5	1	8.18E−04		

rs41282822	20	36869396	G	A	KIAA1755 (blood vessels)	Exonic-synonymous	0.03	0.07	0.02	1	6	1	9.59E−04		
rs41282824	20	36869769	C	T		Exonic-nonsynonymous	0.03	0.07	0.02	1	6	1	9.59E−04		
rs112586932	20	36874313	G	T		Intronic	0.02	0.03	0.02	0	5	1	8.18E−04		

*AF_all, AF_afr, and AF_eur show the allele frequency in all populations, African population, and European population, respectively*.

*Diff_AMR, Diff_CMR, and Diff_NoRej show the number of pairs that are mismatched for antibody-mediated rejection (AMR), T-cell-mediated rejection (CMR), and no-rejection (NoRej), respectively*.

When mapping for eQTLs was performed, 37 eQTLs were found to be enriched in the dataset pertaining to blood vessels and 22 were enriched in the dataset pertaining to whole blood, with several identified “hotspots,” defined by variants that were associated with more than one gene or genes that were associated with more than one variant. Interestingly, we observed that the eQTLs were only found with variants associated with risk of post-tx AMR (Tables S2 and S3 in Supplementary Material).

**Figure 6:**
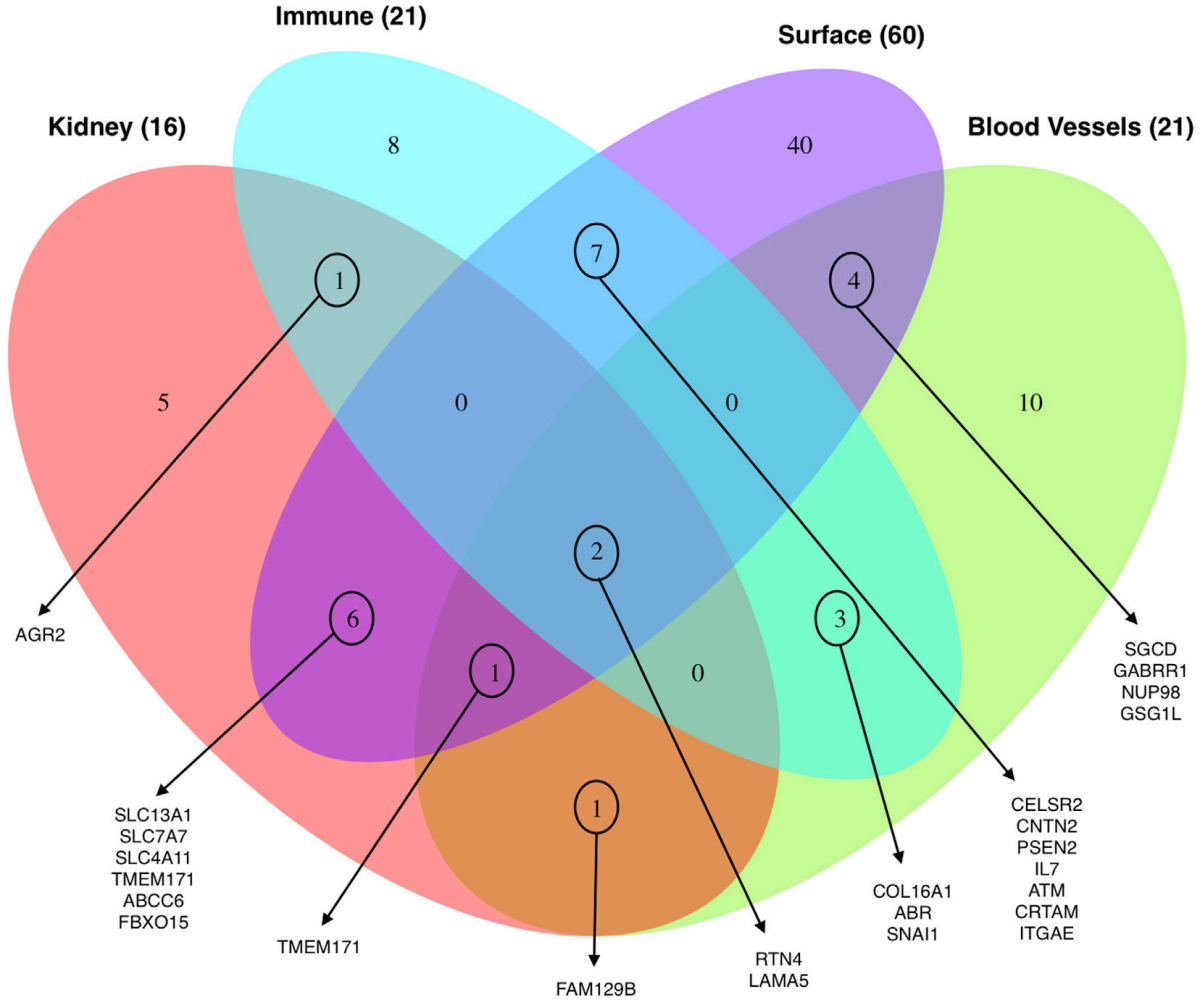
Venn diagram showing the distribution of the total number of genes (98) harboring the 123 significant variants associated with clinical endpoints enriched for their expression in the kidney, blood vessels, immune cells, and cell-surface expression.

Thirdly, we evaluated the enrichment of significant SNPs within four sets of genes, functionally relevant to our study. Leveraging publically available data we evaluated genes that are highly expressed or DE in kidney (the transplanted organ of interest), the endothelium (the target cell of interest in AMR), in immune cells (the effector cells of interest in rejection), and cell surface expressed genes (that may have a higher probability of interaction between the mutated donor antigenic epitope and the recipient antibody paratope). For each set of genes, we performed an enrichment analysis using the χ^2^-test. When considering the variants previously identified to be associated with risk of post-tx AMR, we found statistically significant enrichment in the immune-related genes (*p*-value = 0.007) and cell-surface genes (*p*-value = 4.7*10^−7^). For CMR, we also found significant enrichment for the immune-related genes (*p*-value = 0.02). Figure 6 shows the overlapping genes within each of the four lists of genes.

In addition, variant eQTL analysis in AMR identified significant enrichment for kidney-specific genes (eQTL blood vessels: *p*-value = 0.004; eQTL whole blood: *p*-value = 0.0005) and blood vessels (eQTL blood vessels: *p*-value = 0.02; eQTL whole blood: *p*-value = 0.002).

We finally performed gene set enrichment analysis to find common biological pathways and processes in rejection specific genes that harbor mismatched variants, using the web-based tool EnrichR (42, 43). Importantly, the 72 unique genes associated with AMR were enriched for active transmembrane transporter activity (GO:0022804) (*p*-value = 0.0008) and immune response-activating cell-surface receptor signaling pathway (GO:0002429) (nominal *p*-value = 0.1) corroborating our previous findings. More interestingly, when we assessed the 22 genes that are associated exclusively with CMR, we found enrichment in CD4+ T-cells and CD8+ T-cells (nominal *p*-value = 0.1).

### Machine Learning Techniques Provide a Robust Prediction of Post-tx Rejection Risk Based on Novel D/R Mismatched Variants

**Figure 7:**
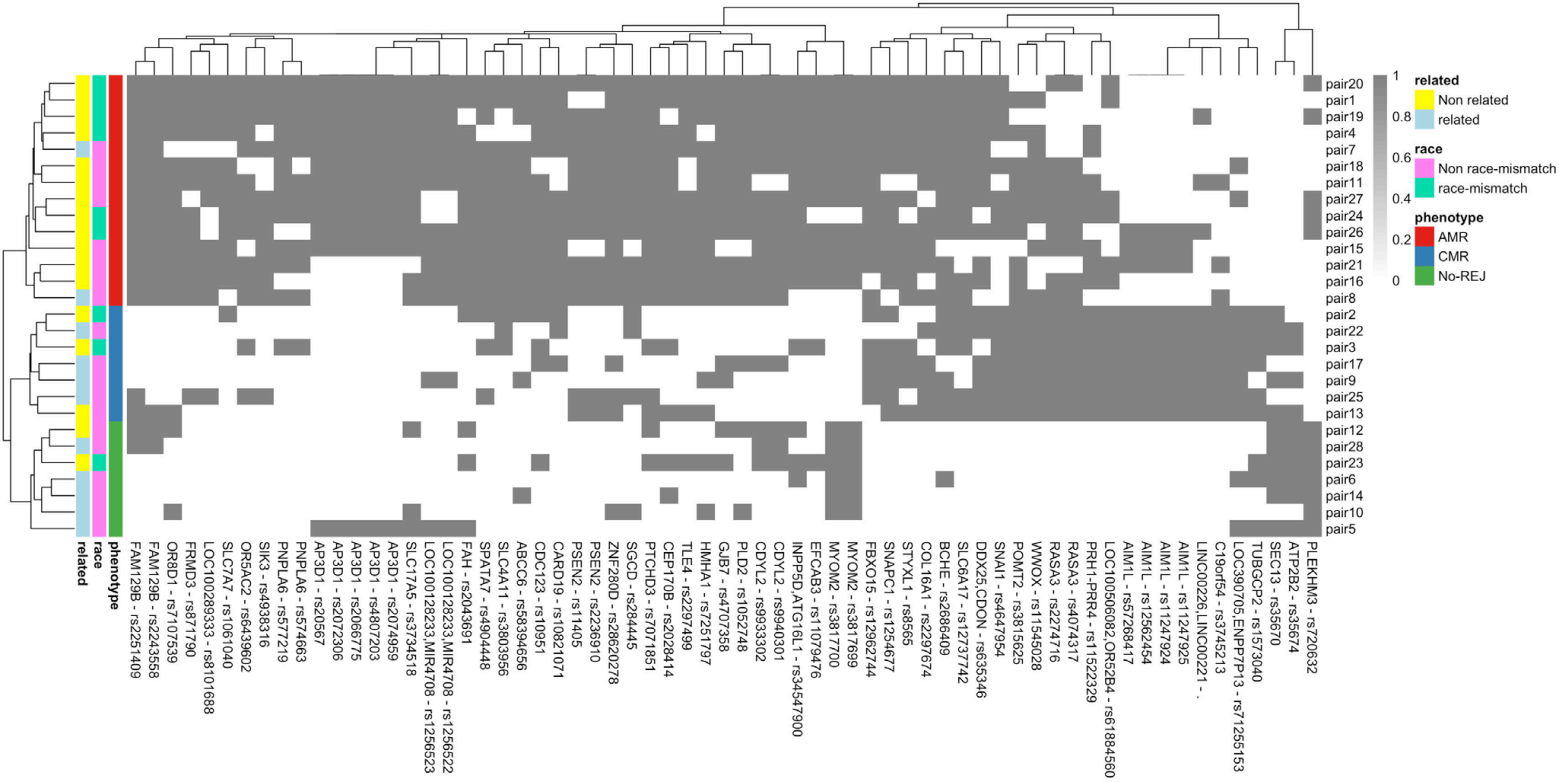
Binary heatmap showing clean separation of the three clinical endpoints of antibody-mediated rejection (AMR), T-cell-mediated rejection (CMR), and no-rejection (NoRej) for the 65 mismatched variants selected by variable selection method using RF. Each gray box represents a variant (*x*-axis) mismatch for that specific D/R pair (*y*-axis), and each white box denotes a variant match between the donor and recipient pair. Each row represents the D/R pairs and columns the variants.

**Figure 8:**
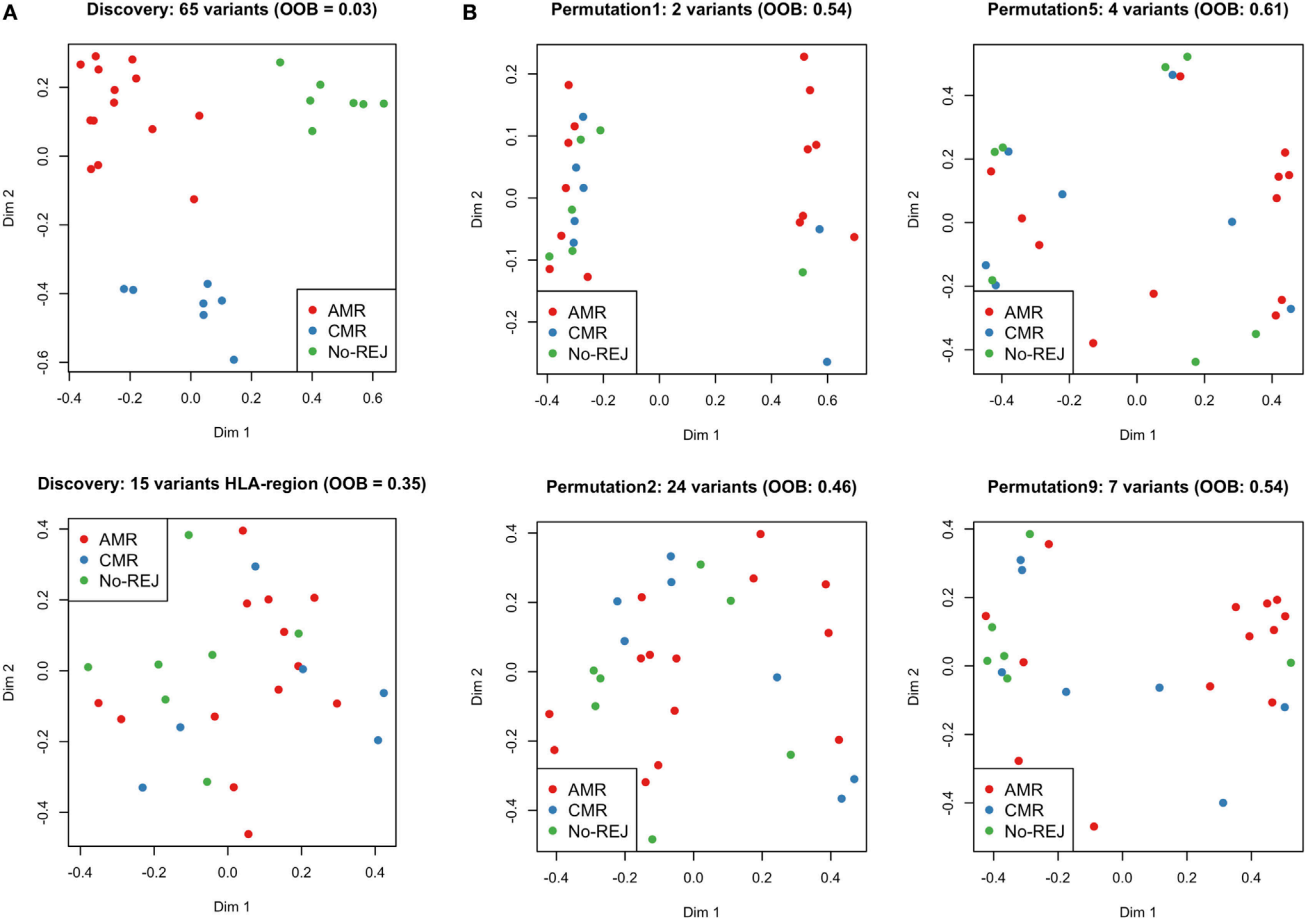
Multi-Dimensional Scaling plots of proximity matrix from RF. **(A)** shows the results from the discovery set applying variable selection method using RF (VSURF) to all the variants mismatched (top) and only to the histocompatibility antigen (HLA) region (bottom) and **(B)** shows the results from the validation set applying VSURF to 10 permuted datasets (only 4 are displayed in the plot).

In the previous analysis using Fisher’s exact test, we analyzed a single variant at a time in a large number of statistical tests, which in combination with the small sample size, resulted in no variants passing the multiple hypothesis correction threshold. Also, modeling a multi-class problem (more than two categories for the clinical endpoints) further adversely influences statistical power. To address this problem, we used a more advanced statistical method to circumvent the issue of statistical power with a machine learning technique, RF. RF builds a classification model for the response variable (clinical endpoints) using all predictors (mismatched variants) quantifying the importance of each predictor. RF by itself does not provide significant levels of individual variants and does not perform variable selection to choose a subset of associated variants, but we were interested to find whether there is a group of mismatched variants that can predict the study clinical endpoints. To this end, we have used a VSURF (37). After applying the VSURF algorithm, we found 65 mismatched variants with a very small OOB error rate (0.03), where OOB measures the accuracy of the final forest. Figure 7 shows the 65 mismatched variants in a binary heatmap, where 1 (gray) represents a mismatch for that particular variant and 0 (white) represents the variant matched in D/R pairs. The three clinical endpoints perfectly cluster together, independent of race mismatch and relatedness, as seen in the color bar on the columns on the left. These variants were also tested with a Fisher’s exact test to find association with race mismatch and relatedness as aforementioned and no significant association was observed. To further verify that our results are not due to random chance, we performed a permutation test shuffling the labels of the clinical endpoints from the original data set. After re-applying the VSURF method, we were no longer able to identify variants that were able to classify the samples. Similar analysis using HLA variants alone could not classify the three different clinical endpoints (average OOB error = 0.25; Figure 8; Figure S4 in Supplementary Material).

## Discussion

Antibody-mediated rejection is a major cause of allograft dysfunction and graft loss as a result of the development of *de novo* DSA to donor-specific HLA antigen mismatches with the recipient after tx (44). The principal targets of the AMR response are the highly polymorphic HLA antigens, but the rejection process has also been observed in HLA-identical siblings (10), suggesting a critical role for D/R nHLA antigen mismatches that may also drive pathogenic antibodies to these mismatched nHLA antigens in AMR (45). Developing methodologies to detect genetic differences between D/R prior to transplant that drive increased AMR risk after tx, will be highly relevant for influencing the long-term outcomes of graft life expectancy after organ tx. This pilot study with carefully selected clinical phenotypes shows a significant increase in the number of mismatched variants prior to tx, which significantly correlate with the development of biopsy-confirmed acute rejection in the recipient after tx.

By developing and applying custom statistical methodologies to exomeSeq data on donor and recipient samples prior to tx, we confirmed our hypothesis that the total number of variants that mismatch by D/R pairs is higher when the recipient goes on to develop AMR after tx. In addition, we also found a highly refined set of variants that can accurately predict immune risk stratification of patients before tx, into those that develop different clinical endpoints after tx of either biopsy-confirmed AMR, biopsy-confirmed CMR or stable function and no rejection. None of these newly identified variants were located in the HLA region, even though the patients involved in this study were sensitized to various HLA antigens, suggesting the possible role of nHLA antigens. Importantly, the AMR group is enriched in race mismatch while NoRej is enriched in relatedness. In all the analysis performed here, these differences have been considered, showing that our findings are independent for both, race mismatch and relatedness.

Further analysis of the 94 variants significantly associated with an increased risk of post-tx AMR located in 72 unique genes are enriched in immune-related function, supporting their role in the rejection process; in addition, these variants also map to genes that are more likely to be expressed on the cell-surface, suggesting that changes in the expression/function of these genes are more likely to be recognized by the recipients’ immune system, and support the possible generation of antibody responses to nHLA targets. These results are supported by a previous study (15), where a cohort with a small number of acute rejections was used to generate an allogenomic mismatch score that associated with transmembrane proteins predicted long-term graft function in kidney transplantation. In this study, we examine a larger number of acute rejections and also stratify risk further by considering both types of acute rejection, AMR and CMR.

We also observe here that specific nHLA variant mismatches impact the development of CMR, as the remaining 25 variants associated exclusively with post-tx CMR. These 25 variants map to 22 unique genes and are highly enriched in immune-related function involving CD4+ T-cells and CD8+ T-cells. This study, thus, also highlights the existence of key intrinsic differences between the triggers and mechanisms of injury in AMR and CMR.

The genes associated with rejection in this study are biologically relevant; specifically those that also have multiple associated variants (*AP3D1, CDC123, CDYL2, CSMD3, FAM129B, MUC3A, MYOM2, OR51F1, OR8G1, OR8G5, PNPLA6, PSEN2, RASA3, ZNF280D, AIM1L, CHRNA10* and *KIAA1755* and SLC-family). 15 out of 18 of these genes associate with risk of post-transplant AMR, and the majority (74%) of non-synonymous variants are located in these genes and in three other genes that associate with the risk of post-tx CMR (*AIM1L, CHRNA10*, and *KIAA1755)*. These variants are likely to be biologically significant for their impact on post-tx rejection. In the follow-up studies we plan to evaluate post-tx nHLA antibody responses to identify the proteins, as we hypothesize that non-synonymous mutated variants produce mutated D/R mRNAs and proteins that can trigger allospecific antibody responses in the recipient and drive AMR. In addition, biological relevance in the context of AMR can be ascribed to many of the identified variants as eQTLs (DNA sequence variants that can influence the expression level of one or more genes) that are significantly enriched in blood vessels and kidney, the target organs of injury in AMR. We observed many hotspots in the endothelial eQTLs where more than one variant is related to one gene and vice versa. For example, the two variants (rs2251409 and rs2243558) located in the *FAM129B* gene are associated with three different genes (*SLC2A8, ZNF79*, and *RPL12)* enriched in the vascular tissues. On the other hand, other genes are associated with multiple variants, e.g., *AP3D1* is associated with five different variants located in the same gene. These eQTLs may be immunologically relevant as they can influence the mRNA and protein expression of multiple genes differentially between donor and recipient pairs and contribute to AMR. Interestingly, all identified eQTLs belong only to variants associated with risk of AMR (and not with risk of CMR) highlighting the importance of detecting these variant differences in D/R pairs prior to engraftment as a means of risk stratification for risk of developing post-tx AMR. The eQTL analysis provide us with an important tool to ascribe relevant functional associations to genes for understanding the process of AMR in organ tx. For example, we highlight that we observe variants in many olfactory transduction factor genes, *OR51F1, OR8G1*, and *OR8G5*, which on initial review, should have no impact on kidney tx outcomes, but more in depth analysis reveals that all these variants also map to an eQTL in blood vessels for a common gene, *VWA5A* (von Willebrand factor A domain-containing protein 5A), which has been shown in a recent study (46), to cause variations in the levels of circulating VWF protein and significantly impact survival after organ tx. Thus, functionally relevant variant differences between donor and recipient may not just relate to mismatched variants in specific genes between the pairs, but may also relate to other downstream genes that these variants may modify.

Though a limitation of this pilot study is the small sample size, we highlight that our study still provides for robust discovery as it benefits from stringent clinical selection criteria for patient selection in each cohort, uses biopsy-confirmed diagnosis for each patient and has extensive statistical data modeling that limits the rate of false positive results. An important analytical caveat in statistical genetics is to control for false positives results, without being too restrictive so as to lose valuable information (false negatives results). Fisher’s exact test is a classic test that does not have enough power to deal with a large number of variables when the sample size is very small and is not ideal for a multi-class problem. To provide for stringent statistical analysis on small sample numbers with many data points, our approach of RF applies a multivariate model (all variables are introduced in the model at the same time) avoiding the correction for MT. The application of VSURF, a strategy that uses the OOB error estimate and the variable importance measures from RF to build an algorithm that performs a variable selection method for each clinical endpoints (AMR, CMR, and NoRej), detects 65 variants, a subset of the 123 variants found with the Fisher’s exact test, that classify all AMR, CMR, and NoRej samples perfectly in regards to patient outcomes after tx. To make our analysis even more stringent, we show that even though RF does not need to correct for MT since it is built using several subsets of random variables, permutation testing further validates our results, as the average OOB error rate from the permuted datasets was 25%, which is quite large in comparison to the one from the discovery set (OOB error = 3%). Thus, a combination of the statistical approaches gives us high confidence in our conclusions that patients who develop AMR after tx have the highest rate of mismatched D/R variants that can be detected before tx. Patients who develop CMR after tx have some shared variants with patients who develop post-tx AMR, which may highlight overlapping mechanisms in both kinds of injury, as previously described (47). Patients who develop CMR post-tx also have unique variants that relate primarily to gene function in CD4/CD8^+^ T cells, the prime cellular player in CMR. The NoRej group is well classified because these patients mostly lack any of the mismatches in the variants in the rejection groups. We recognize that additional independent validation of some of the mutations in the most significant variants is needed.

In conclusion, we have identified a finite and novel set of D/R specific mismatched variants that associate with high risk of rejection after tx and can discern between different histological and prognostic groups of either AMR or CMR after tx. We believe that these variants are functionally relevant as they relate to genes and/or eQTLs that control one or multiple genes that are enriched in the kidney (the organ undergoing injury), are involved in immune function and more likely to be displayed on the surface of the kidney cells, where they can trigger a destructive immune response in the recipient. The current sequencing and custom analytical methodologies can catalog HLA as well as, hitherto undiscovered, nHLA genetic differences between the donor and recipient before tx, that impact clinical outcomes after tx. This critical information can be obtained prior to tx surgery to select an optimal donor when more than one donor is being considered, or to assess post-tx rejection risk of AMR and CMR and personalize induction and maintenance immunosuppression to mitigate immune risk. Preventing rejection, specifically AMR, by optimizing donor selection, would have a significant positive on improving long-term tx outcomes. We believe that the inclusion of a minimal nHLA variant list should be added to current HLA testing to enhance our ability to predict AMR risk, and will fit an unmet clinical need for comprehensive prediction of tx immune risk prior to organ engraftment, opening the door to precision immunosuppression and extended tx survival.

## Ethics Statement

The study is approved by UCSF IRB and the approval number is 14-13573.

## Data and Materials Availability

The exome sequencing data set for this study is available upon request as a collaboration. Please contact Minnie Sarwal at minnie.sarwal@ucsf.edu.

## Author Contributions

MMS, MS, TS, and SP conceived the study design and analysis plan. MS, MMS, and SP carried out the bioinformatics/data analysis plan. SP performed the data analysis and generated all figures and tables. AJ, TS, and MMS collected and analyzed clinical materials. JC contributed to the study design and bioinformatics analyses. MS and MMS supervised the work.

## Conflict of Interest Statement

The authors declare that the research was conducted in the absence of any commercial or financial relationships that could be construed as a potential conflict of interest.

## Footnotes

^1^http://bio-bwa.sourceforge.net/.

^2^http://www.gtexportal.org/home/.

^3^http://www.innatedb.com/.

^4^http://amp.pharm.mssm.edu/Enrichr.

## Supplementary Material

Figure S1 Number of variants mismatched per D/R pair for antibody-mediated rejection (AMR), T-cell-mediated rejection (CMR), and no-rejection (NoRej). W and B represent if the individual in the pair is of Caucasian ancestry/White (W) or African ancestry/Black (B), respectively. The letter at the top denotes the ancestry of the donor and at the bottom is for the recipient. If the pair is related, it is marked with the type of relationship.

Figure S2 Boxplot representing the distribution of mismatched variants in each group per clinical endpoint restricted only to non-synonymous exonic variants **(A)**. Number of variants mismatched per D/R pair for antibody-mediated rejection (AMR), T-cell-mediated rejection (CMR), and no-rejection (NoRej) restricted only to non-synonymous exonic variants **(B)**. W and B represent if the individual in the pair is of Caucasian ancestry/White (W) or African ancestry/Black (B), respectively. The letter at the top denotes the ancestry of the donor and at the bottom is for the recipient. If the pair is related, it is marked with the type of relationship.

Figure S3 On the left panel, boxplot representing the association analysis between histocompatibility antigen (HLA) mismatches and race mismatches for the HLA antigens data **(A)** and HLA exome sequencing (exomeSeq) data **(B)**. On the right panel, boxplot representing the association analysis between HLA mismatches and pair relatedness for the HLA antigens data **(C)** and HLA exomeSeq data **(D)**.

Figure S4 Multi-Dimensional Scaling plots of proximity matrix from RF from the validation set applying variable selection method using RF (VSURF) to 10 permuted datasets.
