# Corrigendum: Novel Non-Histocompatibility Antigen Mismatched Variants Improve the Ability to Predict Antibody-Mediated Rejection Risk in Kidney Transplant

**DOI:** 10.3389/fimmu.2018.00107

**Published:** 2018-01-31

**Authors:** Silvia Pineda, Tara K. Sigdel, Jieming Chen, Annette M. Jackson, Marina Sirota, Minnie M. Sarwal

**Affiliations:** ^1^Division of Transplant Surgery, Department of Surgery, University of California, San Francisco (UCSF), San Francisco, CA, United States; ^2^Institute for Computational Health Sciences, University of California, San Francisco (UCSF), San Francisco, CA, United States; ^3^Department of Medicine, Division of Immunogenetics and Transplantation Immunology, The Johns Hopkins University School of Medicine, Baltimore, MD, United States; ^4^Department of Pediatrics, University of California, San Francisco (UCSF), San Francisco, CA, United States

**Keywords:** kidney organ transplant, antibody-mediated rejection, exome sequencing, non-histocompatibility antigen, gene expression, machine learning

Text Correction

In the original article, there was an error. **The data were intended to be shared in dbGap, but because of some issues in the inform consent, the data cannot be uploaded to dbGap, so we are modifying the way the data will be shared**.

A correction has been made to **Data and Material Availability** and **Paragraph 1**: “The data set for this study will be available at dbGap referenced by the PubMed ID and title of this article.”

for: **“The exome sequencing data set for this study is available upon request as a collaboration. Please contact Minnie Sarwal at minnie.sarwal@ucsf.edu.”**

The authors apologize for this error and state that this does not change the scientific conclusions of the article in any way.

The original article was updated.

## Conflict of Interest Statement

The authors declare that the research was conducted in the absence of any commercial or financial relationships that could be construed as a potential conflict of interest.

