# Supplementary figures and images for: Novel Non-Histocompatibility Antigen Mismatched Variants Improve the Ability to Predict Antibody-Mediated Rejection Risk in Kidney Transplant
Source: Front Immunol. 2017 Dec 5;8:1687. doi: 10.3389/fimmu.2017.01687 (PMC5723302; doi:10.3389/fimmu.2017.01687)

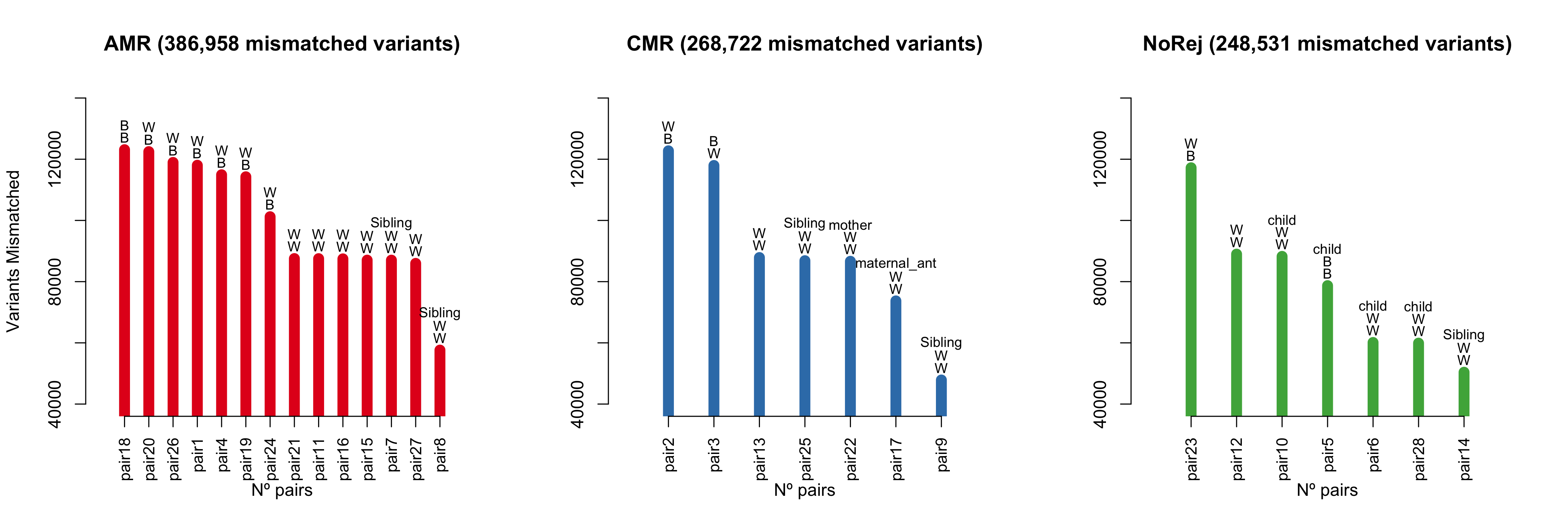

Supplement: Figure S1 — Number of variants mismatched per D/R pair for antibody-mediated rejection (AMR), T-cell-mediated rejection (CMR), and no-rejection (NoRej). W and B represent if the individual in the pair is of Caucasian ancestry/White (W) or African ancestry/Black (B), respectively. The letter at the top denotes the ancestry of the donor and at the bottom is for the recipient. If the pair is related, it is marked with the type of relationship. [file Image_1.jpeg]

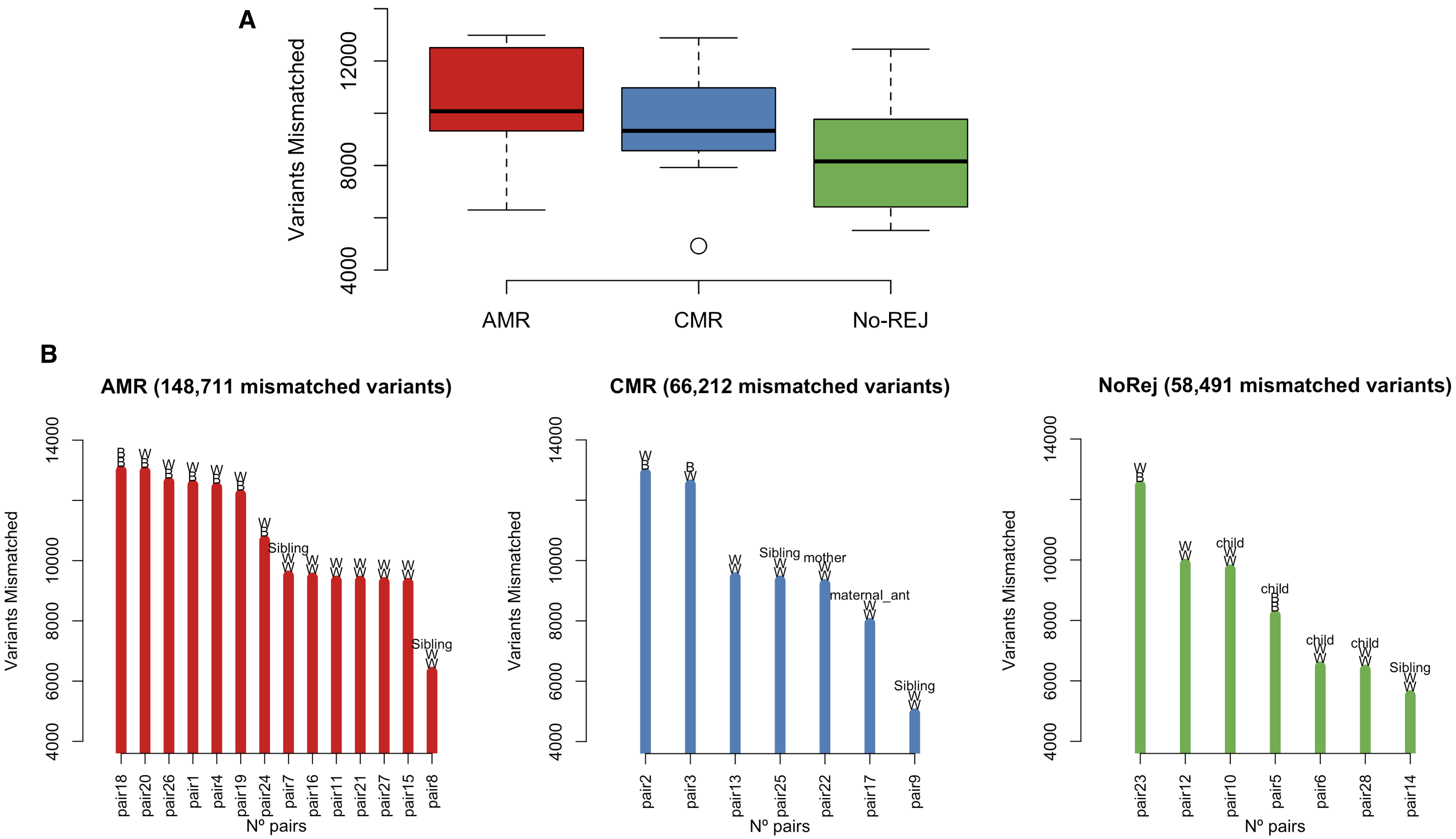

Supplement: Figure S2 — Boxplot representing the distribution of mismatched variants in each group per clinical endpoint restricted only to non-synonymous exonic variants (A). Number of variants mismatched per D/R pair for antibody-mediated rejection (AMR), T-cell-mediated rejection (CMR), and no-rejection (NoRej) restricted only to non-synonymous exonic variants (B). W and B represent if the individual in the pair is of Caucasian ancestry/White (W) or African ancestry/Black (B), respectively. The letter at the top denotes the ancestry of the donor and at the bottom is for the recipient. If the pair is related, it is marked with the type of relationship. [file Image_2.jpeg]

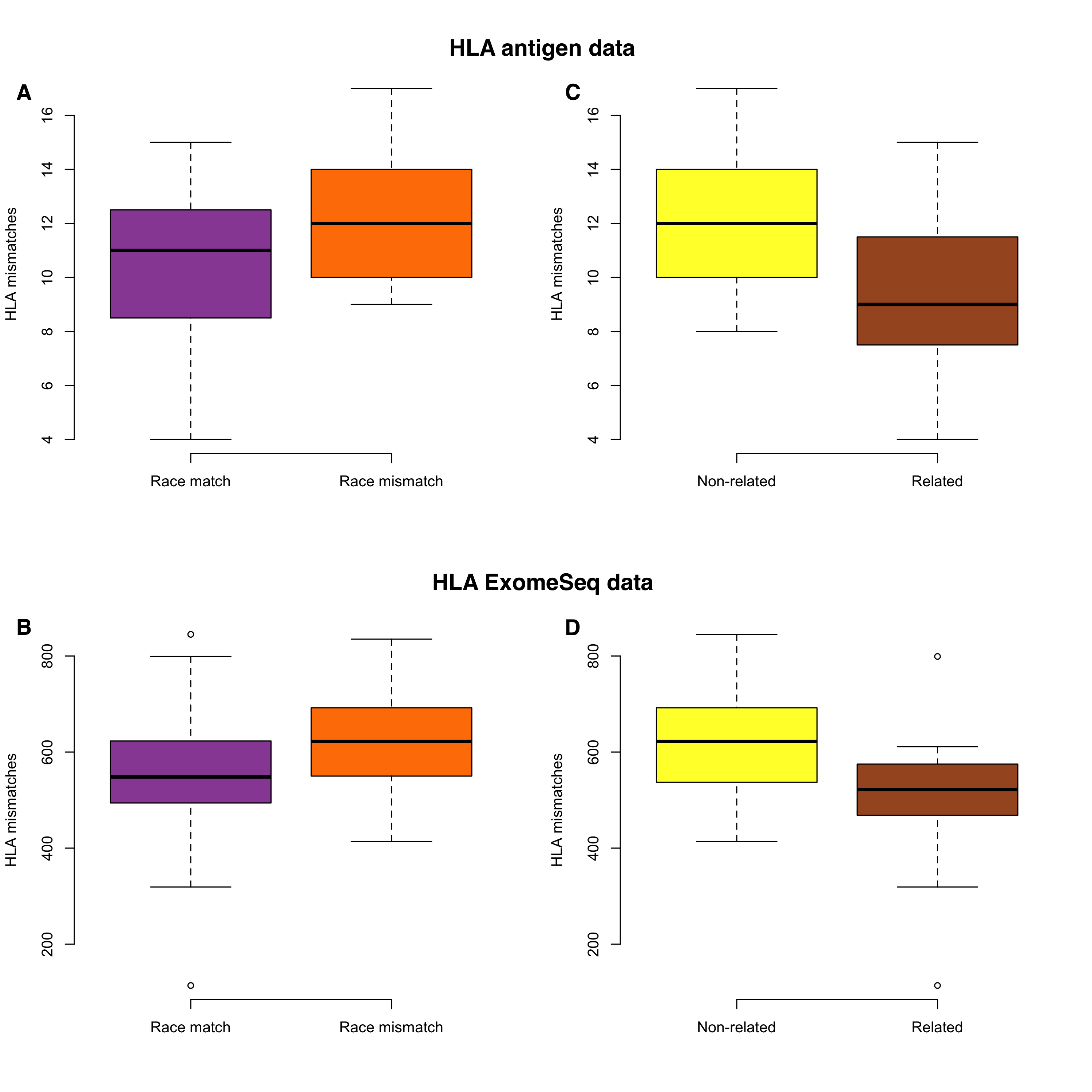

Supplement: Figure S3 — On the left panel, boxplot representing the association analysis between histocompatibility antigen (HLA) mismatches and race mismatches for the HLA antigens data (A) and HLA exome sequencing (exomeSeq) data (B). On the right panel, boxplot representing the association analysis between HLA mismatches and pair relatedness for the HLA antigens data (C) and HLA exomeSeq data (D). [file Image_3.jpeg]

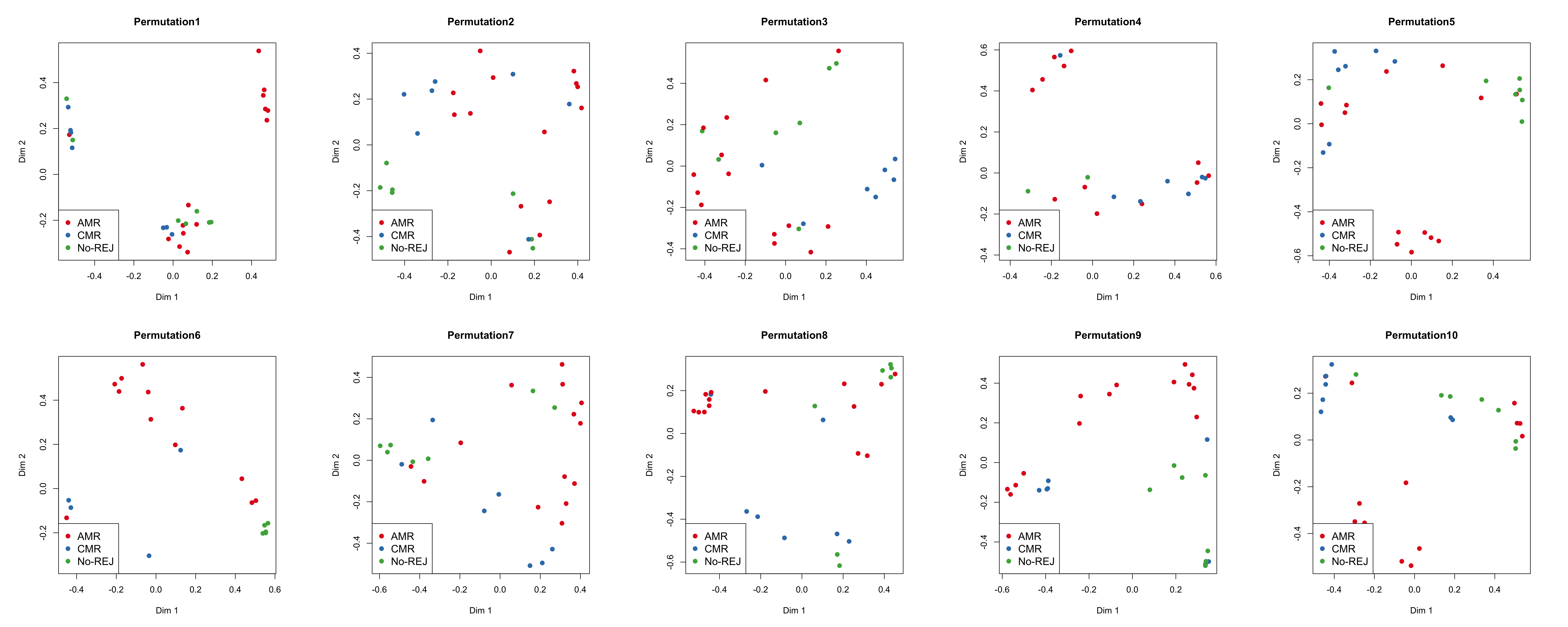

Supplement: Figure S4 — Multi-Dimensional Scaling plots of proximity matrix from RF from the validation set applying variable selection method using RF (VSURF) to 10 permuted datasets. [file Image_4.jpg]
